# Investigating the “Flow” Experience: Key Conceptual and Operational Issues

**DOI:** 10.3389/fpsyg.2020.00158

**Published:** 2020-02-13

**Authors:** Sami Abuhamdeh

**Affiliations:** Department of Psychology, Istanbul Şehir University, Istanbul, Turkey

**Keywords:** flow, enjoyment, task involvement, intrinsic motivation, critical review

## Abstract

The “flow” experience (Csikszentmihalyi, 1975) has been the focus of a large body of empirical work spanning more than four decades. Nevertheless, advancement in understanding – beyond what Csikszentmihalyi uncovered during his initial breakthrough in 1975 – has been modest. In this conceptual analysis, it is argued that progress within the field has been impeded by a lack of consistency in how flow is operationalized, and that this inconsistency in part reflects an underlying confusion regarding what flow is. Flow operationalizations from papers published within the past 5 years are reviewed. Across the 42 reviewed studies, flow was operationalized in 24 distinct ways. Three specific points of inconsistency are then highlighted: (1) inconsistences in operationalizing flow as a continuous versus discrete construct, (2) inconsistencies in operationalizing flow as inherently enjoyable (i.e., “autotelic”) or not, and (3) inconsistencies in operationalizing flow as dependent on versus distinct from the task characteristics proposed to elicit it (i.e., the conditions/antecedents). After tracing the origins of these discrepancies, the author argues that, in the interest of conceptual intelligibility, flow should be conceptualized and operationalized exclusively as a discrete, highly enjoyable, “optimal” state of consciousness, and that this state should be clearly distinguished from the conditions proposed to elicit it. He suggests that more mundane instances of goal-directed engagement are better conceived and operationalized as variations in task involvement rather than variations in flow. Additional ways to achieve greater conceptual and operational consistency within the field are suggested.

## Investigating the “Flow” Experience: Key Conceptual and Operational Issues

Csikszentmihalyi (1975) introduced the concept of “flow” 42 years ago in his groundbreaking book *Beyond Boredom and Anxiety*. The concept of flow was not entirely new – the experience itself held much in common with Maslow’s (1964) conception of “peak experience,” as well as accounts of ecstatic experiences by Laski (1961). However, Csikszentmihalyi’s approach was appreciably more systematic and empirically driven than previous approaches. Within a few years, flow was the focus of hundreds of empirical studies from a diversity of fields including educational psychology, recreation and leisure sciences, game design, and many others.

**FIGURE 1:**
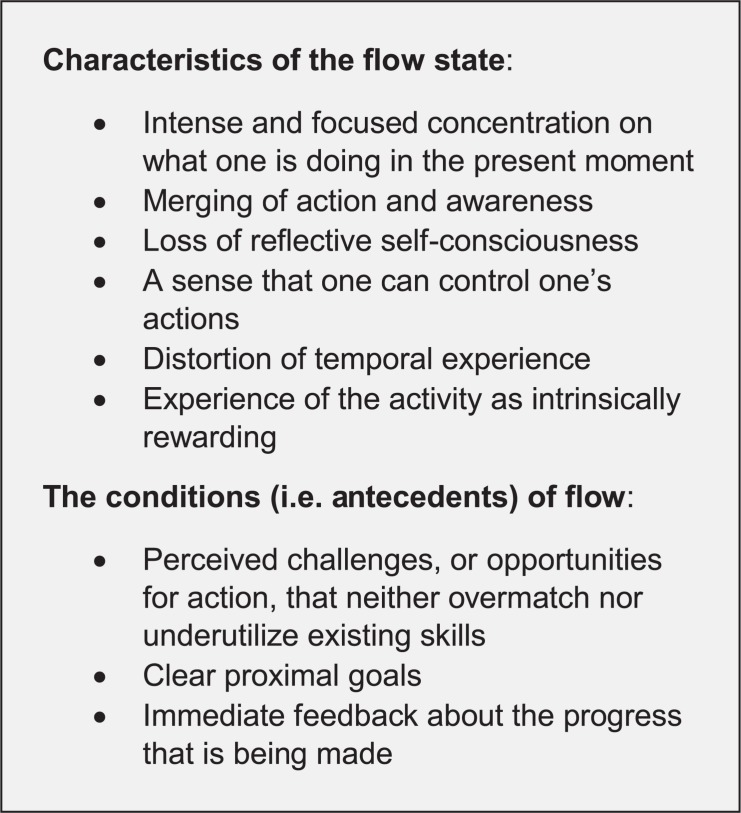
The characteristics and conditions of flow (from Nakamura and Csikszentmihalyi, 2002).

Over the years, many predictors and consequences of “flow”^1^ have been identified (e.g., Jackson and Roberts, 1992; Csikszentmihalyi et al., 1993; Jackson et al., 2001; Demerouti, 2006; Schüler, 2007; Stavrou et al., 2007; Engeser and Rheinberg, 2008; Fullagar and Kelloway, 2009; Nielsen and Cleal, 2010; Bakker et al., 2011; Rodríguez-Sánchez et al., 2011; Seger and Potts, 2012; Coffey et al., 2016). But what have we learned about flow itself – about the state of *optimal experience –* since Csikszentmihalyi introduced the concept in 1975? Here, the view is sobering. The conceptualization introduced in 1975 remains essentially unchanged. Furthermore, fundamental questions persist. [For example, although flow is conceptualized as a multifaceted construct (Figure 1), very little is known regarding its latent structure – the causal relations among its proposed components, the relative contribution of each component to the overall flow experience, etc.]. Indeed, and perhaps most alarming, after almost 42 years of research, there appears to be significant disagreement among researchers regarding what flow actually is and how to measure it. This last point can best be appreciated by first reviewing the many different ways in which flow has been operationalized in the literature.

## A Review of Flow Operationalizations in the Psychological Literature

Within any field of science, the consensual operationalization of central constructs is a *sine qua non* for progress. When this is lacking, results across studies cannot be compared, and the potential for progress in the field is severely undermined. To examine the degree of consistency with which flow has been operationalized within the psychological literature, a review was conducted, limited to publications from the past 5 years^2^. A PsychINFO search yielded the 42 publications listed in Table 1 (see the Appendix for the specific inclusion criteria used to select these publications). As shown in the first column, across the 42 reviewed studies, flow was operationalized in 24 distinct ways. Furthermore, the differences between these operationalizations were often considerable, so that the meaning of “flow” often changed dramatically from one study to the next.

**TABLE 1:** Flow operationalizations in the psychological literature from the past 5 years.

**Flow operationalization**	**Source**	**Continuous or discrete?**	**Enjoyment included?^1^**	**Flow condition(s) included?**
Flow Short Scale (Rheinberg et al., 2003) and related scales^2^	Baumann et al., 2016 Barros et al., 2018 Brom et al., 2017 Harris et al., 2017a, b Hermann and Vollmeyer, 2016 Schattke et al., 2014	Continuous	No	Yes (partly)
Four items from the absorption subscale of the Flow Short Scale	Rivkin et al., 2018	Continuous	No	No
Flow Short Scale with three additional items measuring “autotelic experience”	Rankin et al., 2019	Continuous	Yes	Yes (partly)
Flow State Scale (Jackson and Marsh, 1996) and related scales	Borovay et al., 2019 Beltrán et al., 2018 Forkosh and Drake, 2017 Harmat et al., 2015 Joo et al., 2015 Kaye et al., 2018 Marston et al., 2016	Continuous	Yes	Yes (partly)
As above	Kawabata and Evans, 2016	Discrete	Yes	Yes (partly)
17 of the 36 items in the Flow State Scale	Lin et al., 2019	Continuous	Yes	Yes (partly)
3 of 9 subscales from Flow State Scale	Matthews, 2015	Continuous	No	No
Core Flow Scale (Martin and Jackson, 2008)	Kocjan and Avsec, 2017	Continuous	Yes	No
“3-Channel” flow model (Csikszentmihalyi, 1975)	Huskey et al., 2018 Chen and Sun, 2016 Sun et al., 2017	Discrete	No	Yes (fully)
“Quadrant” flow model (Massimini and Carli, 1988)	Ilies et al., 2017 Sather et al., 2017	Discrete	No	Yes (fully)
Three items measuring interest, enjoyment, and absorption	Bricteux et al., 2017	Continuous	Yes	No
Three items measuring absorption	Vanwesenbeeck et al., 2016	Continuous	No	No
Ten items measuring interest, attention, and control	Cho, 2018	Continuous	Yes	No
Eight items intended to measure conditions and experience of flow	Wanzer et al., 2018	Continuous	Yes	Yes (partly)
Ps presented with description of flow and asked how much their own experience emulated it	Kennedy et al., 2014 Vuorre and Metcalfe, 2016	Continuous	Yes	No
Eight of nine items previously used by Hektner et al. (2007)	Kulkarni et al., 2016	Continuous	Yes	Yes (partly)
A 28 item flow scale (Chou and Ting, 2003)	Soutter and Hitchens, 2016	Continuous	Yes	No
Eleven items taken from Kwak et al. (2014).	Brailovskaia et al., 2018	Continuous	Yes	No
Flow Scale for Games (Kiili, 2006)	Hou, 2015	Continuous	Yes	Yes (partly)
Flow subscale of game engagement questionnaire (Poels et al., 2007)	Dixon et al., 2019	Continuous	No	No
Flow subscale of game engagement questionnaire (Brockmyer et al., 2009)	Smith et al., 2017	Continuous	Yes	No
Three questions prefaced by description of flow (Novak et al., 2000)	Rodríguez-Ardura and Meseguer-Artola, 2017	Continuous	No	No
An 8-item flow scale (Waterman et al., 2003)	Bonaiuto et al., 2016 Mao et al., 2016	Continuous	No	Yes (partly)
Flow questionnaire (Csikszentmihalyi and Csikszentmihalyi, 1988)	Lavoie and Main, 2019	Discrete	Yes	No

*^1^Operationalizations which included one or more items measuring “autotelic experience” (i.e. intrinsically motivating), but did not include items measuring “enjoyment” specifically, were nevertheless classified as having an enjoyment component, given that intrinsic motivation implies enjoyment. ^2^The meaning of “related scales”: We did not distinguish between long versus short versions of scales, nor did we distinguish between older versus newer versions of scales, nor did we distinguish between original versus translated versions of scales. They were all considered to be versions of the same scale and are not differentiated in the table.*

The fourth, fifth, and sixth columns of Table 1 indicate three key ways in which the operationalizations differed. Column 4 indicates whether flow was operationalized as a continuous versus discrete construct in each study. Column 5 indicates whether flow was operationalized as enjoyable (i.e., “autotelic”) or not. Column 6 indicates whether flow was operationalized using one or more of its proposed antecedents (i.e., clear goals, immediate feedback, and a balance of challenge and skill).

In the remainder of this conceptual analysis, I elaborate the nature of the three issues highlighted in Table 1 and attempt to trace their origins. Based on my reading of Csikszentmihalyi’s conceptualization of flow, I suggest that most operationalizations of flow currently found in the literature miss the mark. I argue that flow should be conceptualized and operationalized exclusively as a state of optimal experience – that is, as a discrete, highly rewarding state of consciousness – and that the potential for progress in our understanding of flow largely depends on it.

## The Three Issues

### Issue 1: Is Flow a Discrete or Continuous Construct?

Many psychological constructs, such as happiness, anxiety, and self-efficacy, represent continuous (i.e., spectrum and dimensional) constructs. At any given moment, your happiness may be very low, very high, or anything in between. Other psychological constructs, such as euphoria, fury, and the “suicidal mode” (Rudd, 2000), represent discrete (i.e., categorical and taxonic) constructs. Although it may be possible to locate them on a continuum, they are not applicable to its full range. Occasionally it is not entirely clear whether a construct is continuous or discrete. When this happens in the realm of science, fierce debate usually ensues in an attempt to resolve the conflict. An example of this can be found in the field of abnormal psychology, where the designation of psychological disorders as continuous versus discrete has been hotly contested.

Looking at Column 4 of Table 1, we can see that in a majority of the studies flow was operationalized as a continuous construct, applicable to the full range of participants’ experience in varying degrees. For example, the Flow State Scale-2 (Jackson and Eklund, 2002) composed of items intended to tap the six experiential characteristics of flow, as well as the three conditions (Figure 1), asks participants to indicate the extent to which the items characterize their experience in a just-completed activity on a 5-point Likert scale, ranging from 1 (“strongly agree”) to 5 (“strongly disagree”). Responses to the items are usually averaged to compute a single “flow” score for each and every observation.

**FIGURE 2:**
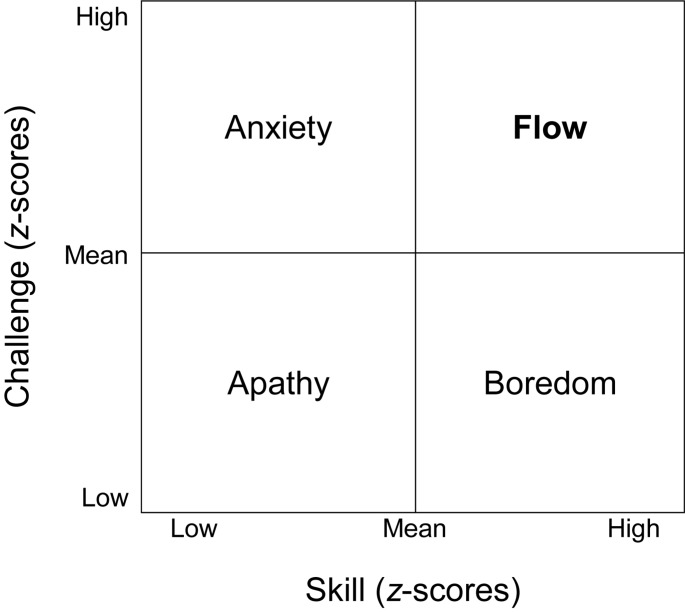
The quadrant model of flow. Challenge and skill scores represent within-person *z*-scores.

A few studies, in contrast, operationalized flow as a discrete construct. For example, two studies which used the experience sampling method (Csikszentmihalyi et al., 1977) used a “quadrant” approach popularized earlier by Csikszentmihalyi and his colleagues (e.g., Csikszentmihalyi and Csikszentmihalyi, 1988; Massimini and Carli, 1988; Figure 2). Using this approach, flow is operationalized as any observation in which both perceived challenge and perceived skill are both “high” (i.e., above the person’s average).

So is flow a continuous construct which exists in greater or lesser degrees across the full range of human experience (like happiness, for example)? Or is it a discrete state that is sometimes experienced, but usually not? In the preface to *Beyond Boredom and Anxiety*, Csikszentmihalyi described flow as such:

“On the rare occasions that it happens, we feel a sense of exhilaration, a deep sense of enjoyment that is long cherished and that becomes a landmark in memory for what life should be like. This is what we mean by “optimal experience.” (p. ii)

Also from the preface:

“From their accounts of what it felt like to do what they were doing, I developed a theory of optimal experience based on the concept of flow – the state in which people are so involved in an activity that nothing seems to matter; the experience itself is so enjoyable they will do it even at great cost, for the sheer sake of doing it.” (p. iv)

As is evident from the passages above (and many others), Csikszentmihalyi conceptualized flow as an “optimal” state of consciousness, one that usually occurs relatively rarely in life. You can be in flow, or not in flow. When you are not in flow, Csikszentmihalyi referred to these states in his work as “non-flow” states (e.g., Csikszentmihalyi, 1975; Csikszentmihalyi and LeFevre, 1989).

Csikszentmihalyi and Csikszentmihalyi (1988) created the Flow Questionnaire as a first attempt to operationalize flow (see Moneta, 2012). Participants are presented with first-hand accounts of what it feels like to be in flow, and then are asked a series of questions including “Have you ever felt similar experiences?” and “If yes, what activities where you engaged in when you had such experiences?” Thus, the Flow Questionnaire operationalizes flow as a discrete construct. Csikszentmihalyi and his colleagues have also used the “quadrant model” (Figure 2) to classify states of consciousness as either flow or non-flow states (i.e., anxiety, apathy, boredom/relaxation) (e.g., Csikszentmihalyi and LeFevre, 1989; Shernoff et al., 2003). This measurement method, too, operationalizes flow as a discrete construct.

Given that Csikszentmihalyi and his colleagues have conceptualized and operationalized flow as a discrete construct, it may be surprising to learn that a significant majority of the studies conducted within the past 5 years operationalized flow as a continuous construct (Table 1). How did this come to be? To address this question, it is necessary to appreciate the difficulty of capturing flow. Flow is described as occurring rarely in regular life (Csikszentmihalyi, 1975, 1990). The rarity with which flow is experienced presents a serious problem for the flow researcher, as statistical power is strongly dependent on having a large sample size. The difficulty of capturing flow is compounded in the psychological laboratory, where participants engage in what is typically an unfamiliar task in an inherently evaluative context. Both of these attributes – the unfamiliarity of the task and the evaluative nature of the context – are likely to work against the (already slim) likelihood of flow being experienced by a study participant, given that (1) flow appears more likely to be experienced by individuals who have developed considerable skill in the activity at hand (Jackson and Csikszentmihalyi, 1999; Rheinberg, 2008; Marin and Bhattacharya, 2013; Cohen and Bodner, 2019) and (2) performance anxiety is not conducive to flow (Csikszentmihalyi, 1975; Fullagar et al., 2013).

One strategy to deal with this “problem of low N” is to reformulate flow from a discrete state of consciousness to one experienced in varying degrees across the full spectrum of conscious experience. Using this approach, any state of consciousness can be classified along a flow continuum, with one end being very low flow and the other end being very high flow (e.g., Jackson and Marsh, 1996; Rheinberg et al., 2003). By doing this, all observations collected in a given study may be included in statistical analyses and contribute toward calculated effects. But reformulating flow in this manner alters the concept in a fundamental way. Flow is by definition an optimal experience, and so designating all other experiences as variations in flow (low flow, moderate flow, etc.) diminishes the intelligibility of the construct. “Low flow” is a contradiction in terms, just as “mild rage” and “moderate ecstasy” are, given that level of intensity is built into the construct.

Besides the conceptual confusion that results from operationalizing flow as a construct applicable to the full range of conscious experience, there is a second reason to avoid operationalizing flow in this manner. When the concept of flow is extended to apply to the full range of experience, it has questionable discriminant validity over pre-existing constructs in surrounding fields. Within the field of intrinsic motivation, dozens of studies have examined a state-level construct called *task involvement* (e.g., Harackiewicz et al., 1987; Elliot and Harackiewicz, 1994, 1996; Tauer and Harackiewicz, 2004; Abuhamdeh and Csikszentmihalyi, 2012a), which represents the degree to which an individual concentrates on and becomes absorbed in an activity. Research on task involvement predates the first operationalizations of flow as a continuous construct, and appears to have been influenced by Csikszentmihalyi’s work on optimal experience (Harackiewicz and Sansone, 1991). If flow is reformulated as a continuous construct, how do we know associated findings are not redundant with what has already been found with respect to task involvement? What is presented as a new contribution to the psychological literature may in fact be old news.

In reality it seems unlikely that there is a sharp boundary between flow and non-flow experiential states. Such thresholds appear to be exceedingly rare when it comes to states of consciousness, even extraordinary ones such as flow. Nevertheless, because flow is conceptualized as an “optimal” experience, it should be operationalized as such. Or else it shouldn’t be called “flow.”

### Issue 2: Is Flow Inherently Enjoyable?

In the preface to *Beyond Boredom and Anxiety* (1975), Csikszentmihalyi described the purpose of his research:

“The goal was to focus on people who were having peak experiences, who were intrinsically motivated, and who were involved in play as well as real life activities, in order to find out whether I could detect similarities in their experiences, their motivation, and *the situations that produce enjoyment.*” (p. xiii)

From this passage, and many others, it is clear that Csikszentmihalyi conceptualized flow as an *enjoyable* experience. Indeed, it was the enjoyable nature of flow, and the positive implications this enjoyment had for motivation, that positioned it as a vehicle for skill development and personal growth (i.e., greater “complexity”) (Csikszentmihalyi and Rathunde, 1998). Csikszentmihalyi hasn’t veered from this initial conception. In more recent work by Csikszentmihalyi and his colleagues, the enjoyable, “autotelic” (i.e., intrinsically rewarding) nature of flow has been consistently emphasized (e.g., Nakamura and Csikszentmihalyi, 2009; Nakamura et al., 2019).

Despite Csikszentmihalyi’s conceptualization of flow as a form of enjoyment, it is quite common for flow researchers to exclude enjoyment (or “autotelic experience”) from their operationalizations of flow, as shown in Table 1. Of the 42 reviewed studies, 17 of them did not include enjoyment (or autotelic experience or intrinsic motivation) in their operationalizations. How did this come to be? Why is flow being operationalized by some flow researchers without an enjoyment component? In reviewing the history of this issue I identified several likely sources (Abuhamdeh, in press).

#### Source #1: Martin Seligman

Beginning in his bestselling book *Authentic Happiness* (2002), Seligman (2011) began asserting that “it is the absence of emotion, of any kind of consciousness, that is at the heart of flow.” (p. 111). Seligman (2011)’s reasoning for this is expressed in many places, including his modestly titled follow-up book *Flourish: A Visionary New Understanding of Happiness and Well-being* (2011), in which he wrote: “I believe that the concentrated attention that flow requires uses up all the cognitive and emotional resources that make up thought and feeling.” (p. 11).

Judging by how often he has been cited, flow researchers have taken Seligman’s views on flow very seriously. But his assertion that flow is devoid of emotion is in direct conflict with Csikszentmihalyi’s conceptualization of flow as a form of enjoyment (given that enjoyment is an emotion). Furthermore, the notion that the intensive allocation of cognitive resources to a task prevents emotions from being experienced is at odds with contemporary emotion theory and research. Perhaps the most complete account of how emotions are elicited is provided by appraisal theories of emotion (Arnold, 1960; Lazarus, 1966; Scherer, 1984; Smith and Ellsworth, 1985; Frijda, 1986; Oatley and Johnson-Laird, 1987). Among appraisal theorists, there is consensus that appraisals do not always require conscious intervention (Ellsworth and Scherer, 2003; Moors, 2010). In fact it is generally presumed that appraisal processes usually occur automatically (Smith and Kirby, 2001; Moors, 2010). Appraisals must be fast and efficient given that changes in the environment can occur very quickly (Lazarus, 2001). Thus, like other automatic processes, they need not consume significant attentional resources.

Appraisal theorists also agree that with increasing practice there is greater automatization of appraisal processes (Moors et al., 2013). This has particular relevance for flow because flow appears to be more commonly experienced by individuals who are quite skilled in the activity they are engaged in (and thus have logged many hours of practice) (Csikszentmihalyi, 1975; Dietrich, 2004; Marin and Bhattacharya, 2013; Cohen and Bodner, 2019). Therefore, it seems especially likely that any appraisal processes that may occur during flow are mostly or fully automatic.

#### Source #2: A Failure to Differentiate Between *Experiencing* Emotions and One’s *Awareness and Labeling* of These Emotions

One defining feature of flow is an absence of self-awareness. Flow researchers have sometimes assumed that this absence of self-awareness during flow prevents the experience of emotion during flow. For example, from a recent paper (Kyriazos et al., 2018): “Flow-ers seem to be almost beyond experiencing emotions, probably due to the absence of self-awareness…” But self-awareness is not a precondition for the *experience* of emotions, only the *recognition* and *labeling* of them. This is why non-human mammals who lack a sense of self are nevertheless capable of experiencing emotions (Panksepp, 2005). Similarly, among humans, those younger than 7 months (and who therefore have not yet developed a sense of self) are nevertheless able to experience a wide range of emotions (Izard et al., 1995). The only emotions not in the repertoire of these children appear to be the so-called “self-conscious emotions” (e.g., pride, shame, and guilt), which young children first appear capable of experiencing between the ages of 2.5 and 3 years (Lewis, 2008). Indeed, even children who lack a cerebral cortex are capable of experiencing emotions (Merker, 2007).

#### Source #3: Csikszentmihalyi’s Confusing Usage of the Word “Pleasure” in His Work

In his book Flow (1990), Csikszentmihalyi wrote, “None of these [flow] experiences may be particularly pleasurable at the time they are taking place, but afterward we think back on them and say, “That really was fun” and wish they would happen again.” This statement may seem to imply that the experience of flow itself may not be particularly enjoyable. However, to properly interpret this passage it is necessary to understand Csikszentmihalyi’s unusual usage of the word “pleasure” in his work, and the sharp distinction he draws between pleasure and enjoyment. Csikszentmihalyi (1990) considers pleasurable experiences to be those that satisfy biological needs, such as eating and sleeping (p. 45). According to Csikszentmihalyi, the experience of pleasure is derived from “restorative homeostatic experiences.” Thus an artist who stayed up all night feverishly working on a painting, foregoing both food and rest, did not have a “pleasurable” experience according to Csikszentmihalyi’s usage, because the behavior did not satisfy any biological needs (in fact it was in conflict with them). But this should not be misinterpreted as implying that the artist did not enjoy him/herself.

### Issue 3: Should Flow Be Partly or Fully Operationalized Using Its Proposed Antecedents?

Csikszentmihalyi and his colleagues make a clear distinction between the conditions of flow and the experience of flow itself (Figure 1). Yet if we refer once again to Table 1, we see that a large number of studies ignored this distinction by operationalizing flow using *both* the experiential elements of the flow state and one or more of the conditions of flow. For example, in the Flow State Scale (Jackson and Marsh, 1996), some items measure the experiential elements of flow (e.g., “I had total concentration”) whereas others measure the proposed conditions (e.g., “my goals were clearly defined”). The items are then usually averaged by researchers to yield a single “flow” score.

Given the strong distinction Csikszentmihalyi and his colleagues make between the conditions proposed to elicit flow and the state of flow itself, why is this distinction routinely ignored in empirical work? One explanation may be found in Csikszentmihalyi’s earlier work. Though for the past several years Csikszentmihalyi and his colleagues have drawn a sharp distinction, this was not always the case. In *Beyond Boredom and Anxiety* (1975), for example, Csikszentmihalyi himself grouped the conditions of flow with the experiential elements by including all of them under the heading “Elements of the flow experience” (p. 38). And this continued for several years. In *Flow* (1990), he included both the conditions of flow and the experiential elements under the general heading “The elements of enjoyment.” (p. x). It wasn’t until approximately 20 years ago that Csikszentmihalyi and his colleagues began consistently differentiating the conditions from the experience.

Additionally, it should be noted that Csikszentmihalyi and his colleagues themselves sometimes operationalized flow based solely on the ratio of challenges and skills (e.g., Massimini and Carli, 1988; Csikszentmihalyi and LeFevre, 1989; Stein et al., 1995; Shernoff et al., 2003; Asakawa, 2004). Indeed, before the current popularity of flow scales, this was the most common way to operationalize flow. This likely served to further reinforce the idea that flow and the conditions that elicit it are one and the same.

So how to proceed? It has been argued that the primary objective of any scientific endeavor is to provide causal explanations (e.g., Popper, 1957; Shadish et al., 2002). Thus the conceptual distinction Csikszentmihalyi and his colleagues make between the conditions of flow and the state itself is an important one. Indeed, much of what distinguished Csikszentmihalyi’s initial work on flow from previous work on peak experiences was that he attempted to not only describe the experience, but to explain it by identifying the conditions which elicited it. This is why Csikszentmihalyi’s work on flow is sometimes referred to as a “model” or “theory.” Without distinguishing cause from effect, however, it is neither.

That the distinction should be consistently made is supported by empirical findings, too. “Flow” (as measured by the Flow Short Scale, Rheinberg et al., 2003) is not always optimized by a balance of challenges and skills, which suggests that inferring flow based on this condition is not a safe bet (Engeser and Rheinberg, 2008). Indeed, the relationship between challenge and enjoyment appears to be very unstable across both activity and person (Abuhamdeh and Csikszentmihalyi, 2009, 2012b). This variation helps account for why the variance in subjective experience explained by challenge-skill ratios across all daily activities tends to be low (Ellis et al., 1994).

As can be seen in Table 1, most of the commonly used flow scales conflate the conditions and the experience. One notable exception among them, however, is the 10-item Core Flow Scale (Martin and Jackson, 2008), used in one of the 42 studies. The aim of the scale, as described by the authors, is “to assess the central subjective (phenomenological) experience of flow.” Because this scale does not conflate the conditions of flow with the experience of flow, it may be the best option among the current fleet of validated scales. However when using this scale, or any other which purports to measure the components of flow, it is advisable to allow the weighting of the components to vary freely rather than the usual custom of assuming they are equal and taking their average, since the relative contribution of each component to the overall experience of flow in specific contexts is unknown (see Jackson and Marsh, 1996).

## Two Remaining Questions

The preceding discussion raises two specific questions which deserve to be addressed here.

### Question 1: If Flow Is to Be Operationalized as a Discrete Construct, Where Should the Boundary Between “Flow” and “Non-flow” Be Set?

This is clearly a difficult question to answer satisfactorily.^3^ A sharp boundary or threshold is unlikely to exist. Individuals who describe their optimal experiences do not commonly report a sudden transition point between flow and non-flow. This therefore presents a dilemma for the flow researcher, as any delineation of a cutoff would necessarily involve a degree of arbitrariness. Nevertheless, to remain true to flow’s conceptualization as a discrete state, a boundary must be set.

Previous attempts to distinguish flow from non-flow have varied considerably in approach. The most common approach has been to classify experience based on challenge–skill ratios (such as the quadrant model shown in Figure 2). However, this approach infers flow based solely on a single proposed condition (the balance of challenge and skill), which, as previously discussed, is not warranted. Furthermore, dividing experience in such a manner often results in 25% or more of all daily experiences being designated as “flow” experiences (e.g., Csikszentmihalyi and LeFevre, 1989; Hektner and Asakawa, 2000).

Rather than the researchers deciding which experiences qualify as flow experiences, an alternative strategy has been to have the participants decide for themselves. Indeed, this is how Csikszentmihalyi initially began measuring flow experiences (see Moneta, 2012). In the Flow Questionnaire (Csikszentmihalyi and Csikszentmihalyi, 1988) respondents are first provided with a description of a flow experience, and then are asked to indicate whether they have ever experienced flow. If so, various follow-up questions about these experiences are then asked. Similar measures which tap single flow experiences have since been created (e.g., Novak et al., 2003). These measures appear to come closest to operationalizing flow as it is conceptualized – as a discrete, optimal state of consciousness. Unfortunately, they are not commonly used. Out of the 42 studies listed in Table 1, only one used such a measure.

Kawabata and Evans (2016), noting the inability of most commonly used flow scales to differentiate flow experiences from non-flow experiences (e.g., the Flow State Scale, Jackson and Marsh, 1996; the Flow Short Scale, Rheinberg et al., 2003), proposed a remedy. They first administered one of the more popular flow scales to participants (the Flow State Scale-2; Jackson and Eklund, 2002) immediately following physical activity of some sort (e.g., physical education class and training session). They then used latent class analysis to divide participants into four groups based on the participants flow scores. Kawabata and Evans noted that the participants in the two groups with the highest item-averages both had average scores greater than 3 (the midpoint of the 5-point scale), and on this basis they proposed that the participants in the two groups experienced flow. This constituted 54% of the sample. Though the sensibility of the criterion used in this case to delineate a cutoff appears dubious and resulted in a suspiciously high number of participants who were deemed to have experienced flow, the study represents the first serious attempt to rectify what is a major limitation of most flow scales.

Although no sharp boundary between “flow” and “non-flow” is likely to exist, this does not mean that a cutoff cannot be based on sensible criteria. This may seem contradictory, but such cut-offs are routinely designated for practical reasons in other fields, with success (for example in the medical sciences for high blood pressure, obesity, etc., as well as in clinical psychology for the assessment of psychological disorders). Taxonomic analytic techniques (Meehl, 1995; De Boeck et al., 2005; Ruscio et al., 2006) appear especially well-suited for identifying potential cut-off points. As one possibility, previous factor analyses based on data derived from flow scales indicate that two of the proposed components of flow – a lack of self-consciousness and a merging of action and awareness – load poorly on a higher-order “flow” factor (see Swann et al., 2018), even though these two features were commonly mentioned features of flow in Csikszentmihalyi’s early interviews. One possible explanation for this is that these two features only become experientially salient at very high levels of involvement, which may have been underrepresented in the factor-analytic studies. If this is the case, the implied inflection point would offer a sound basis for a cut-off. More generally, taxonomic analytic techniques should help clarify whether flow represents a difference in quality of experience versus simply a difference in degree.

### Question 2: What About “Sub-Optimal” Experiences? Does the Flow Model Have No Relevance for Them?

In this conceptual analysis I’ve argued that flow should be operationalized as Csikszentmihalyi conceptualized it: as an exceptional, “optimal” experience. But what about less intense, “non-flow” states of goal-directed engagement? Does the flow model have no relevance when it comes to these much more common states? Clearly it does. There is evidence that all three of the proposed antecedents of flow (clear goals, immediate feedback, and optimal challenges), in at least some situations, promote enjoyment (Harter, 1978; Reser and Scherl, 1988; Abuhamdeh and Csikszentmihalyi, 2012b; Pratt et al., 2016). But the fact that the conditions of flow have relevance for these states should not prompt researchers to automatically label these states as flow, as doing so obfuscates the meaning of flow.

It is interesting to note that Csikszentmihalyi himself recognized the relevance of the flow model for less intense states than flow. He introduced the concept of “micro-flow” to help account for such experiences (Csikszentmihalyi, 1975). However, the introduction of another discrete construct (with all the accompanying operational dilemmas) to account for less intense states at this point seems unnecessary. Two pre-existing constructs in the motivation literature, mostly ignored by flow researchers, appear very capable of capturing such states. Crucially, both of them are continuous constructs that can be applied meaningfully to the full range of conscious experience.

#### Construct #1: Task Involvement

Flow has been described as being composed of cognitive, emotional, and motivational components (e.g., Delle Fave and Massimini, 2005). In terms of its cognitive aspect, the defining feature of flow is intense attentional focus on the task at hand (Nakamura and Csikszentmihalyi, 2002). It is this deep attentional involvement that appears to underlie several of the other characteristics of flow including the merging of action and awareness and the absence of self-consciousness (Dietrich, 2004; Csikszentmihalyi et al., 2005; Kawabata and Mallett, 2011).

Task involvement, as previously described, represents the degree to which an individual concentrates on and becomes absorbed in an activity (Elliot and Harackiewicz, 1994). Operationalizations usually include items that measure both absorption and concentration. The task involvement construct nicely captures the central cognitive feature of flow. In contrast to flow, however, task involvement is a purely cognitive phenomenon representing the degree of attentional involvement in an activity; it is not inherently enjoyable and motivating in concept, though it often predicts both (Abuhamdeh and Csikszentmihalyi, 2012a).

#### Construct #2: Intrinsic Motivation

Because of the enjoyable nature of flow, it is “autotelic,” meaning it motivates the person who experiences it to continue doing what he/she is doing. The meaning of autotelic and intrinsic motivation are synonymous. Intrinsic motivation, as conceptualized and operationalized within the motivation literature, captures both the emotional and (therefore) motivational properties of flow, yet, in contrast, is applicable to the full range of conscious experience.

The standard way to measure intrinsic motivation is by asking participants how *enjoyable* and *interesting* the activity they are (or were) engaged in is. The measurement of both enjoyment and interest is important, because interest appears to be a positive emotion distinct from enjoyment (Tomkins, 1962; Izard, 1977; Panksepp, 1998; Silvia, 2008). This view is backed by empirical findings which indicate that interest and enjoyment, in at least some contexts, have different antecedents, as well as different trajectories in response to performance feedback (Reeve, 1989; Egloff et al., 2003).

In sum, the conditions of flow have implications for a much wider array of states than just flow. The constructs *task involvement* and *intrinsic motivation* appear particularly well-suited for capturing these states. The incorporation of these constructs into empirical investigations of goal-directed engagement has the added benefit of allowing the associated research findings to be more easily assimilated into the surrounding motivation literature.

## Summary and Conclusion

Almost 50 years ago, Csikszentmihalyi (1975) began a program of research with the aim of understanding the common experiential characteristics of so-called “optimal experiences,” as well as the conditions which promote these experiences. To this end, he asked hundreds of rock climbers, chess players, artists, etc. to describe what their best moments felt like. Based on this research, Csikszentmihalyi developed the concept of “flow.”

Since that time, hundreds of empirical studies have been conducted in an attempt to further understand flow. Yet if we survey the ways in which flow has been operationalized in these studies, we are forced to reckon with an unsettling fact: a consensual operationalization of flow has yet to be established. Across studies, operationalizations vary considerably, so that the meaning of flow from one study to the next often changes drastically.

In this conceptual analysis, I’ve highlighted three key inconsistencies found in flow operationalizations: (1) inconsistences in operationalizing flow as a discrete versus continuous construct, (2) inconsistencies in operationalizing flow as inherently enjoyable (i.e., autotelic) or not, and (3) inconsistencies in operationalizing flow as dependent on versus distinct from the task characteristics proposed to elicit it (i.e., the conditions/antecedents). I’ve argued that these inconsistencies are born out of conceptual misunderstandings, as well as the methodological difficulties inherent in operationalizing optimal experience.

The lack of a standard operationalization of flow does not bode well for the field. It is only by adopting a standard operationalization that questions about the nature of flow (e.g., is the distortion of time a consistent component of optimal experience?) as well as flow’s relation to other constructs (e.g., what is the relationship between flow and performance?) can be addressed. It is only by the consistent application of a standard operationalization that a period of “normal science” (Kuhn, 1962) may ensue.^4^

Given that a standard operationalization of flow is needed, whose conceptualization of flow should it be based on? A tacit assumption made throughout this paper is that Csikszentmihalyi’s conceptualization of flow is the only valid conceptualization. The reasoning for this is as follows: Unlike most psychological constructs, which are generic in their nature (e.g., euphoria, misery, anxiety, etc.), we put “flow” in quotes (or italicize it, or write it with a capital F) because it is a proper noun, a term coined by a specific psychologist to represent his particular conceptualization of optimal experience. In other words, the term flow comes with Csikszentmihalyi’s conceptualization “pre-installed.” His conceptualization is therefore the default conceptualization, and this is true regardless of its merits.^5^

Of course, once this conceptualization is operationalized in a valid and consistent manner, and systematically tested and evaluated, it may turn out that Csikszentmihalyi’s conceptualization of optimal experience should be modified or updated in one or more ways. In this case, a revised conceptualization would be warranted. This would be a positive development, a sign of progress.

## Author Contributions

The author confirms being the sole contributor of this work and has approved it for publication.

## Conflict of Interest

The author declares that the research was conducted in the absence of any commercial or financial relationships that could be construed as a potential conflict of interest.

## Footnotes

1 Here I put “flow” in quotes because, as will be shown, most studies of flow haven’t operationalized flow as conceptualized by Csikszentmihalyi – as a (discrete) state of optimal experience.

2 Thanks to Şahika Dilgüşa Durmuş, Khaled Mahmoud Elazab, and Selenay Keleş for their help with this review.

3 The difficulty this presents is one reason why, in my own empirical work on goal-directed engagement, despite my longstanding interest in flow, I’ve resisted operationalizing flow altogether, instead opting to measure experience in a more piecemeal fashion using lower-level constructs that can be meaningfully applied to the full range of conscious experience (e.g., Abuhamdeh and Csikszentmihalyi, 2012a; Abuhamdeh et al., 2015).

4 Swann et al. (2018) recently assessed the current state of flow research in sport and exercise psychology, using Kuhn’s (1962) model of scientific development as a guide. Their provocative thesis was that flow research, following a long period of “normal science,” is now approaching a “crisis point.” However in Kuhn’s (1962) scheme, “normal science” represents the practice of working within a firmly established research paradigm, characterized by, among other things, uniform conceptualizations and standard operationalizations. As shown in the current paper, flow research cannot be characterized as such. At least from a methodological standpoint, the current state of the field seems to have more in common with the preceding stage in Kuhn’s (1962) scheme – what he referred to as the “pre-paradigm” stage. Indeed, in his famous book, Kuhn (1962) himself seemed to imply that all of the social sciences are pre-paradigmatic (p. 161).

5 By the same token, if I formulated a conceptualization of ecstatic love which I called Glow, and other researchers, inspired by my work on Glow, wished to investigate it, they would need to operationalize Glow as I conceptualized it (as a state of ecstatic love) in order to make any claims about Glow based on their subsequent findings.

## Appendix

### How the Publications in Table 1 Were Selected

An “advanced search” in PsycINFO specified the following parameters:

(1) Publication date: July 2014 to July 2019.
(2) Publication type: Peer-reviewed journals.
(3) Subject: Major heading: Flow (consciousness state).

This yielded 111 publications. Forty-one of these publications did not include a flow operationalization, and were therefore not included in the review. Of the remaining 70 publications, those which included one or more of the following features were also not included in the review:

(1) Publications in which flow was operationalized as a trait-level construct (e.g., “flow proneness”) rather than a state-level construct.
(2) Publications in which flow was operationalized as “collective flow.”
(3) Publications in which the flow operationalization was not clearly described.
(4) Publications in which flow was operationalized in two or more distinct ways.
(5) Publications not in English.
(6) Validation studies.

This process yielded the 42 publications shown in Table 1. Although not exhaustive (given the inclusion criteria above), the listing is intended to be adequately representative of the operationalizations found in the psychological literature.
